# Long-Term Follow-Up Cares and Check Initiative: A Program to Advance Long-Term Follow-Up in Newborns Identified with a Disease through Newborn Screening

**DOI:** 10.3390/ijns10020034

**Published:** 2024-04-18

**Authors:** Mei Lietsch, Kee Chan, Jennifer Taylor, Bo Hoon Lee, Emma Ciafaloni, Jennifer M. Kwon, Megan A. Waldrop, Russell J. Butterfield, Geetanjali Rathore, Aravindhan Veerapandiyan, Arya Kapil, Julie A. Parsons, Melissa Gibbons, Amy Brower

**Affiliations:** 1 American College of Genetics and Genomics, Bethesda, MD 20814, USA; mlietsch@acmg.net (M.L.); kchan@acmg.net (K.C.); jtaylor@acmg.net (J.T.); 2 Department of Neurology, University of Rochester, Rochester, NY 14627, USA; bohoon_lee@urmc.rochester.edu (B.H.L.); emma_ciafaloni@urmc.rochester.edu (E.C.); 3 Department of Neurology, University of Wisconsin School of Medicine and Public Health, Madison, WI 53726, USA; kwon@neurology.wisc.edu; 4 Center for Gene Therapy, Abigail Wexner Research Institute, Nationwide Children’s Hospital, Columbus, OH 43205, USA; megan.waldrop@nationwidechildrens.org; 5 Department of Neurology and Pediatrics, Ohio State University Wexner Medical Center, Columbus, OH 43210, USA; 6 Department of Pediatrics and Neurology, University of Utah, Salt Lake City, UT 84132, USA; russell.butterfield@hsc.utah.edu; 7 Division of Neurology, Department of Pediatrics, University of Nebraska Medical Center, College of Medicine, Omaha, NE 68198, USA; grathore@childrensomaha.org; 8 Division of Neurology, Department of Pediatrics, University of Arkansas for Medical Sciences, Arkansas Children’s Hospital, Little Rock, AR 72202, USA; aveerapandiyan@uams.edu (A.V.); karya@uams.edu (A.K.); 9 Department of Pediatrics, University of Colorado School of Medicine, Aurora, CO 80045, USA; julie.parsons@childrenscolorado.org (J.A.P.); melissa.gibbons@childrenscolorado.org (M.G.); 10 Genetic Medicine, University of Nebraska Medical Center, Omaha, NE 68198, USA

**Keywords:** newborn screening (NBS), long-term follow-up (LTFU), spinal muscular atrophy (SMA)

## Abstract

In the United States and around the world, newborns are screened on a population basis for conditions benefiting from pre-symptomatic diagnosis and treatment. The number of screened conditions continues to expand as novel technologies for screening, diagnosing, treating, and managing disease are discovered. While screening all newborns facilitates early diagnosis and treatment, most screened conditions are treatable but not curable. Patients identified by newborn screening often require lifelong medical management and community support to achieve the best possible outcome. To advance the long-term follow-up of infants identified through newborn screening (NBS), the Long-Term Follow-up Cares and Check Initiative (LTFU-Cares and Check) designed, implemented, and evaluated a system of longitudinal data collection and annual reporting engaging parents, clinical providers, and state NBS programs. The LTFU-Cares and Check focused on newborns identified with spinal muscular atrophy (SMA) through NBS and the longitudinal health information prioritized by parents and families. Pediatric neurologists who care for newborns with SMA entered annual data, and data tracking and visualization tools were delivered to state NBS programs with a participating clinical center. In this publication, we report on the development, use of, and preliminary results from the LTFU-Cares and Check Initiative, which was designed as a comprehensive model of LTFU. We also propose next steps for achieving the goal of a national system of LTFU for individuals with identified conditions by meaningfully engaging public health agencies, clinicians, parents, families, and communities.

## 1. Introduction

In the United States (US), newborn screening (NBS) is a multi-component, multi-stakeholder public health system designed to identify infants with serious disorders whose outcomes can be improved by early identification and treatment [1]. Ideally, NBS should begin with prenatal education of expectant parents, followed by population-based screening of newborns and referral of newborns at risk for a NBS condition to clinical specialists. These clinical specialists can then perform confirmatory testing and clinical examination to establish diagnoses, and then treat and manage the diagnosed individuals [1,2]. All components of this multi-step system are necessary to ensure newborns who are identified with a condition achieve the best possible outcomes. The number of screened conditions in the NBS system has increased rapidly, triggered by discoveries of novel technologies for screening, diagnosing, treating, and managing disease. This has led to an expansion in the number and variety of clinical specialists who receive referrals of newborns at risk for an NBS condition [1,2]. State public health laboratories have had to expand their efforts to assess and coordinate the follow-up and treatment of at-risk newborns with a broader range of clinical specialists than pediatricians and geneticists, e.g., immunologists, endocrinologists, and neurologists. This network of care emphasizes early diagnosis and timely treatment, with some public health data being collected about these activities in the initial months of life. However, even though most infants will continue to require ongoing care and management throughout their lives, there are no consistent mechanisms for collecting longer term data. Partnerships between clinicians, state public health agencies, parents, families, and communities are essential to ensure children identified as having or being at risk for a heritable condition through NBS are healthy, growing, and thriving [3].

To facilitate long-term follow-up (LTFU) efforts, the Health Resources and Services Administration (HRSA) Maternal and Child Health Bureau (MCHB) created the Long-term Follow-up for Severe Combined Immunodeficiency and Other Newborn Screening Programs (LTFU Program). The purpose of this program is to support comprehensive models of LTFU that demonstrate collaborations between clinicians, public health agencies, and families. In 2021, the HRSA awarded a two-year cooperative agreement to the American College of Medical Genetics and Genomics (ACMG) to extend the LTFU Program. The Long-Term Follow-up (LTFU) Cares and Check Initiative was developed to design, implement, and evaluate data collection and reporting tools with a focus on newborns identified with spinal muscular atrophy (SMA) through NBS. LTFU themes which inform parents and families of health information and outcomes were prioritized, while data collection and visualization tools were made available to pediatric neurologists who care for newborns with SMA. The data tracking and visualization tools were delivered to state NBS programs collaborating with a pediatric neurologist associated with a clinical referral center. In this publication, we report on the development, use of, and preliminary results from the LTFU-Cares and Check Initiative, which was designed as a comprehensive model of LTFU. We also propose next steps for achieving the goal of a national system of LTFU for individuals with NBS conditions that meaningfully engages public health agencies, clinicians, parents, families, and communities.

## 2. Methods

### 2.1. Use Case

SMA was chosen as the use case because it is recommended that treatment of newborns diagnosed with SMA occurs within the first weeks of life, and this timeline of early intervention requires close collaboration between neuromuscular providers, e.g., pediatric neurologists or physiatrist, and state NBS programs [1,4]. The recommended NBS screen detects the common deletion in the survival motor neuron 1 (*SMN1*) gene [5]. The treatment and clinical management of SMA requires additional laboratory-based testing to confirm the deletion in the *SMN1* gene and determine the SMN2 copy number [5]. *SMN2* is a partially functional copy of *SMN1* determining the severity of the disease [5]. The SMN2 copy number is not part of the RUSP recommendation, and most state NBS programs do not routinely include SMN2 copy number in screening [5]. Increasingly, follow-up staff of state NBS programs help to coordinate this testing as a second-tier test [5]. While NBS for SMA coupled with the new therapies represents a significant breakthrough, clinicians and state NBS programs are facing new challenges. Among these challenges are determining the optimal timing for early intervention and selecting appropriate treatment options, including the consideration of combination therapies [5]. This LTFU program presents a promising model to address these challenges.

### 2.2. Program Goals

1. Engage parents and families living with newborns, children, and adults with SMA to identify LTFU themes to prioritize for SMA;
2. Design, implement, and evaluate data collection with input from at least five pediatric neurologists caring for NBS-identified SMA cases;
3. Connect the clinical site with their respective state NBS program to foster LTFU collaboration;
4. Design, implement, and evaluate data dashboards designed for clinical sites and state NBS programs;
5. Design and disseminate LTFU data points that are important for the LTFU of SMA to all state NBS programs in the US;
6. Increase the number of infants, children, and families who receive coordinated LTFU care through a medical home by 20 percent from baseline.

### 2.3. Workflow

**Figure 1:**
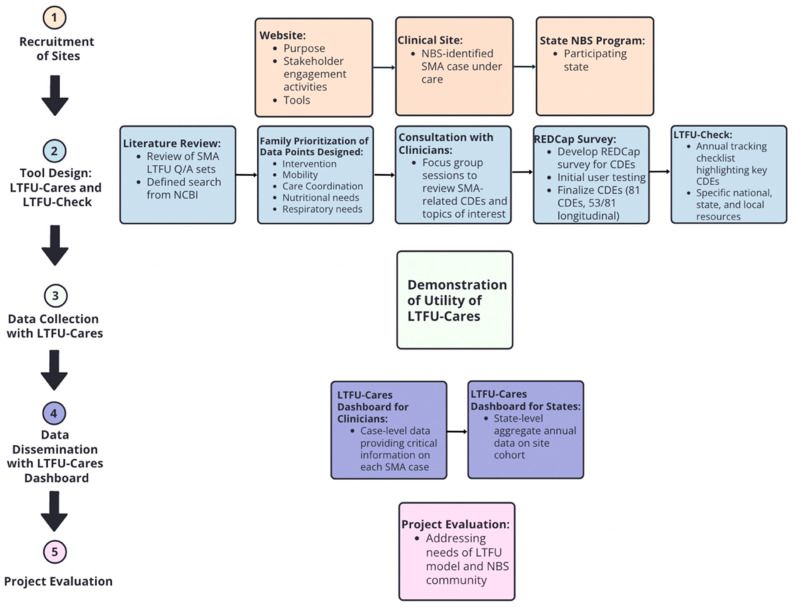
LTFU-Cares and Check Initiative Workflow based on program goals.

Figure 1 shows the LTFU-Cares and Check Initiative Workflow based on the five program goals: (1) Recruitment of Sites, (2) Tool Design, (3) Data Collection with LTFU-Cares, (4) Data Dissemination with LTFU-Cares Dashboard, and (5) Project Evaluation.

1. Recruitment of Sites

Website

The LTFU Cares and Check Initiative utilized a multi-method approach to engage stakeholders across the NBS lifecycle of screening, diagnosis, clinical care, and long-term management with a focus on individuals, institutions, and state NBS programs involved in SMA NBS. In October 2022, we launched the website https://longtermfollowupnbs.org (accessed on 12 April 2024), where we describe the purpose, stakeholder engagement activities, and the tools in development: LTFU-Cares, LTFU-Check, and the LTFU Cares Dashboard. In 2023, we measured the website’s analytics to evaluate the visibility of the tools.

Clinical Sites

We leveraged the Newborn Screening Translational Research Network (NBSTRN) Steering Committee and Clinical Integration Group (CIG) to identify pediatric neurologists who care for individuals with NBS-identified SMA. We invited the pediatric neurologists to collaborate and guide the development of data points for the LTFU-Cares data collection tool, enter data on at least one SMA case identified through NBS, collaborate with their respective state NBS program, and provide feedback on the LTFU of NBS conditions. These states covered 14.5% of the births in the US [6] and had the potential to detect over 200 cases of SMA per year.

State NBS Programs

Based on the engaged clinical sites, we invited state NBS programs to collaborate, provide input on the LTFU-Cares data collection tool, receive a LTFU-Cares Dashboard, receive a LTFU-Check for SMA, and provide feedback on the LTFU of NBS conditions.

2. Tool Design

Literature Review

The development of the LTFU-Cares data collection tool included a review of two established SMA LTFU Q/A sets by ACMG staff, clinical experts, and state NBS programs. To refine the scope of the Q/A sets, also known as common data elements (CDEs), a targeted search on the National Center for Biotechnology Information (NCBI) at the US National Library of Medicine (NLM) was conducted. This literature review aimed to incorporate recent research findings and ensure that the CDEs were current and aligned with recent advancements in SMA research. The search was limited to articles related to spinal muscular atrophy and treatments published from 2018 to 2022 and human age groups from birth to five years of life [5,7,8,9,10,11,12,13,14,15,16,17,18,19,20,21].

Family Prioritization of Data Points

We used a two-phased approach to solicit input from parents of children with SMA to guide the development of the LTFU data tools and resources. Input from parents and families was expected to inform overall definitions of “medical home”, “LTFU”, and “care coordination”. Four families with NBS-identified SMA children were engaged in a discovery process using contextual interviews. The interviews were designed to be open-ended, with the families sharing their experiences and thoughts about LTFU for SMA. The topics and concepts discussed by the families were combined with keywords provided from literature, key effort reviews of SMA, and key words from the ACMG team. In phase two, the resulting list of 150 topics was distributed to the SMA community via a social media platform, and participants were asked to choose a limited number of topics that were important to them and SMA LTFU. This exercise provided a picture of family priorities in SMA LTFU and was used to inform the development of the LTFU Cares data collection tool. The prioritized topics were utilized to evaluate the overall project.

Consultation with Clinicians

The topics derived from the literature review and those identified as priorities by SMA families were reviewed in four focus group sessions. Five pediatric neurologists were recruited to review these topics based on their active involvement in the study and their expertise in SMA.

LTFU-Cares Using REDCap^®^

The refined list of CDEs was provided to clinicians through the Research Electronic Data Capture (REDCap^®^) web application. REDCap^®^ is widely used by academic, public health, clinical, and research teams to collect health information [22,23,24,25,26,27]. Clinicians provided an initial user testing of the tool, and feedback from this testing phase was collected. Modifications were made to improve the usability of the data set. A finalized minimal dataset containing 81 CDEs, 65% of which were longitudinal (53/81), was developed including clinical site information, subject details from newborn screening results and confirmatory testing, health outcomes, treatments, and social determinants of health affecting care coordination [28].

LTFU-Check

**Figure 2:**
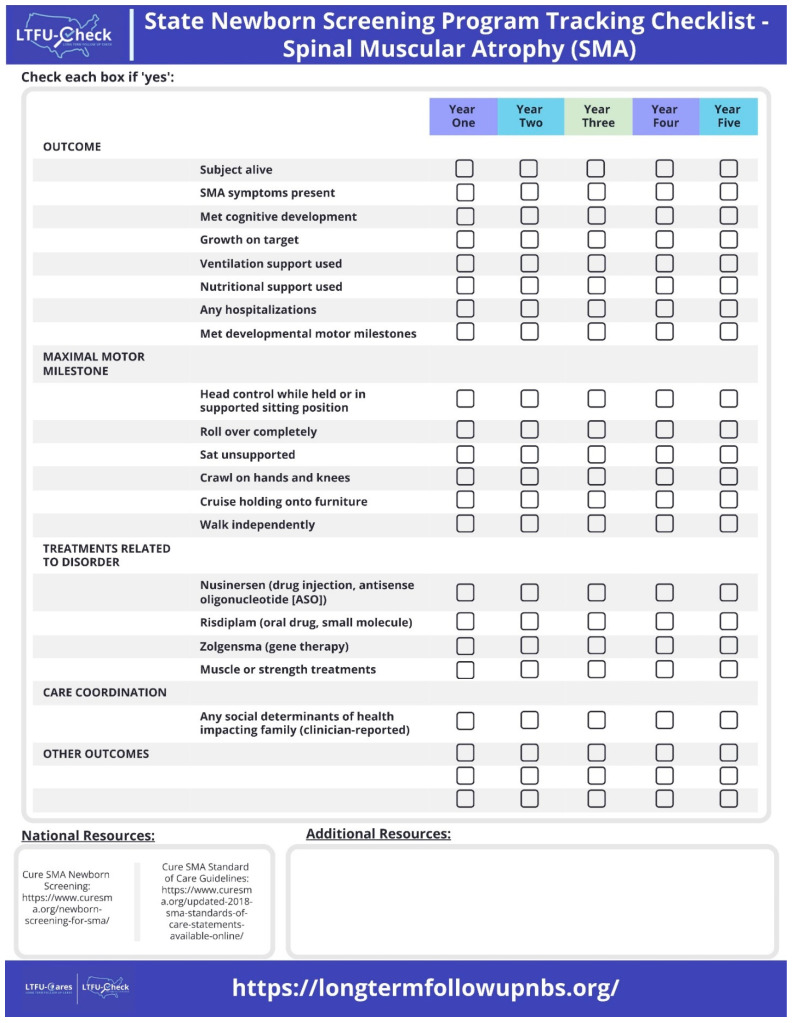
LTFU-Check Reporting and Monitoring Checklist. The LTFU-Check provides annual checklists for outcomes, treatments, and care coordination with an additional resources section.

Figure 2 displays the LTFU-Check Reporting and Monitoring Checklist. The LTFU-Check provides annual checklists for outcomes, treatments, and care coordination with an additional resources section. The LTFU -Check was designed to meet the specific needs of state NBS programs, and the content was based on input from the clinical sites and their prioritiztion of CDEs within the minimal dataset that were most informative for an annual tracking checklist. Within the LTFU-Check tool, national [29,30], state, and local resources tailored to meet the needs of each individual participating state NBS program were provided based on clinical recommendations. All measurements related to outcomes are reported by clinicians based on their individual practice.

3. Data Collection using LTFU-Cares

Clinicians were asked to enter data on at least one SMA case with up to five years of annual data in the REDCap^®^ survey since NBS of SMA began in July 2018 [31]. There was no limit to the number of cases clinicians could enter. Pediatric neurologists reviewed all data within each reported year of life, aggregating and summarizing information rather than entering data for individual visits. Eligible cases included all newborns encountered by the pediatric neurology clinic with SMA identified through NBS. Cases younger than the age of one year and not identified with SMA through NBS were excluded from data collection.

4. Data Dissemination with LTFU-Cares Dashboard

Venngage was used to present data visualization models showcasing de-identified case-level data for clinicians and aggregate annual state-level data for state NBS programs. Both dashboards included breakdowns of demographic information, newborn screening results, health outcomes compared to a baseline [32,33], interventions, and care coordination based on the data collected. Data presented in this dashboard were only shared with the reporting clinical site and its respective state NBS program.

5. Project Evaluation

To evaluate the model’s usability and adaptability for other NBS conditions, a multifaceted approach was used. Feedback was actively collected from clinicians and state NBS programs through surveys, a three-part discussion, and email communications. All challenges encountered and project recommendations were documented. In addition, project materials were disseminated to participating state NBS programs to serve as a foundation for future NBS projects.

## 3. Preliminary Results

### 3.1. Recruitment of Sites

#### 3.1.1. Website

From September 2022 to January 2024, our platform attracted a total of 499 users, with 66% (328/499) being new users who recently registered or accessed the site for the first time. Moreover, 31% (157/499) of users expressed interest in learning about the project, while 19% (93/499) were interested in the data page designed as a resource for participating clinicians and state programs. During this time, we saw a spike in user engagement on the website by at least 200% following marketing promotions (such as published articles (ACMG Ezines and ACMG Medical Geneticist) and conference presentations). From September to January 2024, 8% (22/499) of website users demonstrated an active interest in joining our efforts.

#### 3.1.2. Clinical Sites

Eight pediatric neurologists who care for NBS-identified SMA cases at seven hospitals were engaged. The clinicians participated in multiple focus group sessions to design the LTFU-Cares data collection tool (81 data elements, 65% being longitudinal (53/81)), advise on the LTFU-Check data fields, and provide feedback on the design of the LTFU-Cares data dashboard to encourage data use and sharing with the state NBS program. In addition, they all agreed to be involved in communications with their state NBS programs to facilitate care coordination and health outcome tracking. Each site entered retrospective, longitudinal data on at least one case. The number of cases from each site ranged from one to nine. The range of time points collected for each case ranged from one to five years of life.

#### 3.1.3. State NBS Programs

All states with a clinical site agreed to participate (New York, Arkansas, Colorado, Nebraska, Ohio, Wisconsin, and Utah). All state NBS programs in the US will receive an electronic copy of LTFU-Check (Figure 2) as part of our dissemination of the project.

### 3.2. Tool Design

#### 3.2.1. Parent and Family Engagement

Sixteen families were enrolled in this phase of the project. Of these families, 100% (16/16) had a child with SMA, 50% (8/16) of whom were identified through NBS. Moreover, 75% (12/16) of the survey respondents were female, and 25% (4/16) were male. A total of 62.5% (10/16) of respondents reported an annual income above USD 100,000. The children of five of sixteen (31.25%) respondents had the most severe type of SMA, called SMA Type 1. Five topics were prioritized by all the respondents, listed in rank order: #1 Gene Therapy; #2 Improved Outcomes for Child; #3 Improving Outcomes for Families; #4 Mobility Issues and Progression; and #5 Time to Initial Treatment.

There were differences in the prioritized topics between the NBS-identified families and the non-NBS-identified families. For the non-NBS-identified families, mobility issues and progression, pulmonary concerns, muscle health, assistive technology, and prognosis were the top five topics. This may reflect an older cohort of children or a more advanced disease. Mobility issues were less of a priority for families whose children had milder forms of SMA, i.e., Type 3 and Type 4. Most families reported that they were currently receiving specialty SMA care, as detailed in the following table. The degree and frequency of specialty care varied greatly from family to family and did not correlate with SMA type or any other queried demographic. No families characterized the care their child was receiving using the words “medical home”, but all families expressed a strong relationship with their child’s neurologist.

An online survey operationalized by LunaDNA asked participants to choose a limited number of important topics, resulting in a very accurate picture of patient priority around a large variety of topics shaping their experiences. In total, 87.5% (14/16) families had insurance of some kind, with over 50% having multiple types of insurance coverage (private, Medicaid, government assistance, employer-provided). Transcripts from the interviews were reviewed using natural language processing (NLP) to identify the needs of the families. Most families reported that their physical needs were being met, but only a small percentage reported that their emotional needs were being met. Parents reported that the most significant gap in having their needs met occurred around the transition from pre-birth and pre-diagnosis to diagnosis and treatment. The family engagement component of our project represented a single point in time; therefore, a determination of the percentage of infants lost to follow-up is not possible. The LTFU-Cares component is expected to provide information on patients lost to follow-up across the seven clinical sites.

#### 3.2.2. LTFU-Cares Data Collection

A total of 81 CDEs were incorporated in data collection, with 65% (53/81) of CDEs specifically used for longitudinal tracking. Of these, 46% (37/81) CDEs were required, and 86% (6/7) sites completed all required questions. The clinical site information (11%, 9/81 CDEs), subject details (16%, 13/81 CDEs), demographics (7%, 6/81 CDEs), health outcomes (41%, 33/81 CDEs), and treatment (12%, 10/81 CDEs) were the most complete forms of CDE. The care coordination (12%, 10/81 CDEs) was the least complete. One site (14%, 1/7) did not complete the care coordination form. Of the data collected, a total of 18 cases were entered by clinical sites. All cases (18/18) have data for the first year of life, a fraction have data at different time points beyond the first year, 33% (6/18) have data from two years, and 17% (3/18) have three years of data. Clinicians were required to enter data on a minimum of one case, but 86% (6/7) of sites entered data for more than one case.

#### 3.2.3. LTFU-Cares Data Dashboard

**Figure 3:**
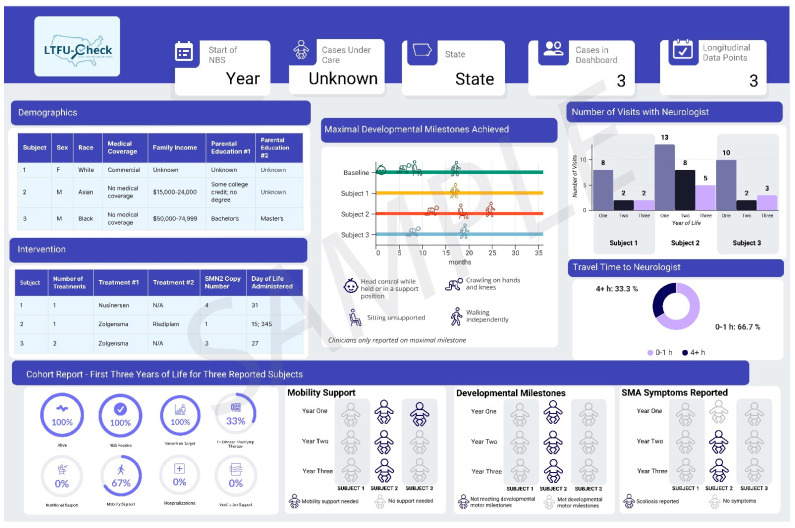
Sample dashboard for clinical sites.

**Figure 4:**
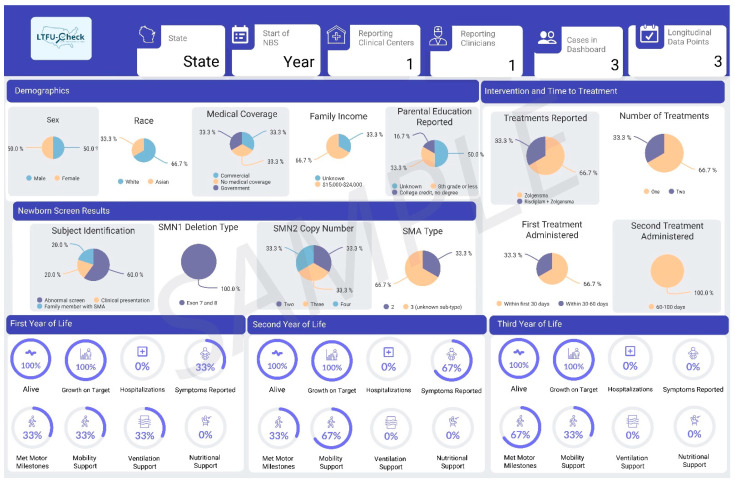
Sample dashboard for state NBS programs.

Results from the LTFU-Cares Dashboard showcase case-level data tailored to pediatric neurologists (Figure 3) and state-level data for the respective states (Figure 4). Each dashboard is customized based on the data collected from LTFU-Cares. The design of the dashboard was shaped by collaborative consultations with clinicians who provided input on the information they wanted featured in the dashboards. All clinicians (100%) emphasized the importance of including maximal motor milestones achieved over time, a feature tailored to help them assess if cases were on target, as shown in the middle of Figure 3. Additionally, clinicians expressed a need to include detailed information on the timing of treatment from the NBS result on state dashboards, which is shown on the right of Figure 4. Key information, including demographics, newborn screening results, and cohort reports, were included in both dashboards.

## 4. Discussion

We successfully developed and implemented the LTFU Cares and Check Initiative to design, implement, and evaluate data collection and reporting tools with a focus on newborns identified with SMA through NBS. Future efforts will evaluate the results of the program and compare the LTFU themes that were prioritized by parents and families with the data points of interest to pediatric neurologists and state NBS programs. Gathering input from all stakeholders is important to identify the disease-specific and general data points that are most helpful to facilitate care coordination, inform evidence-based treatment, enable continuous quality improvement, and contribute to new knowledge discovery. If the project had additional time, gathering more input for the minimal data set would have been helpful. The LTFU-Cares and Check Initiative is a helpful model of LTFU that provides a system of meaningful engagement between state NBS programs and the clinicians who care for individuals identified with an NBS condition. Although our project focused on SMA, the methodology and tools can be adapted to all conditions that are part of, or candidates for, NBS. Importantly, our project facilitates the HRSA MCHB goal of LTFU for individuals with NBS conditions that meaningfully engages public health agencies, clinicians, parents, families, and communities.

## Data Availability

The data that support the findings of this study are available upon request from the corresponding author (A.B.).
